# Spatiotemporal characteristics of the pharyngeal teeth in interspecific distant hybrids of cyprinid fish: Phylogeny and expression of the initiation marker genes

**DOI:** 10.3389/fgene.2022.983444

**Published:** 2022-08-16

**Authors:** Qianhong Gu, Hui Yuan, Hui Zhong, Zehong Wei, Yuqin Shu, Jing Wang, Li Ren, Dingbin Gong, Shaojun Liu

**Affiliations:** ^1^ The State Key Laboratory of Developmental Biology of Freshwater Fish, College of Life Sciences, Hunan Normal University, Changsha, China; ^2^ Guangdong Laboratory for Lingnan Modern Agriculture, Guangzhou, China

**Keywords:** *in situ* hybridization, *scpp5*, pharyngeal teeth, phylogeny, expression pattern, distant hybridization

## Abstract

As an important feeding organ and taxonomical characteristic, the pharyngeal teeth of cyprinid fish have very high morphological diversity and exhibit species-specific numbers and arrangements. Many genes have been verified to regulate the pharyngeal teeth development and act as the initiation marker for teeth. Six initiation marker genes for pharyngeal teeth were used as RNA probes to investigate the expression pattern, and these genes were further used to construct a phylogenetic tree for cyprinid fish including some distant hybrids. The results from *in situ* hybridization showed that similarities and differences existed in the expression of *dlx2b*, *dlx4b*, *dlx5a*, *pitx2, fth1b*, and *scpp5* in the pharyngeal region of the hybrids (BT) by the crosses of blunt snout bream (BSB, ♀) × topmouth culter (TC, ♂). Particularly, we found a high specificity marker gene *scpp5* for the early development of pharyngeal teeth. The *Scpp5* expression pattern established a clear graphic representation on the spatiotemporal characteristics of the early morphogenesis of pharyngeal teeth in BT and BSB. Our results suggested that the s*cpp5* expression in 4V^1^, 3V^1^, and 5V^1^ in BT occurred earlier than that in BSB, while the replacement rate of pharyngeal teeth (4V^2^, 3V^2^, and 5V^2^) was faster in BSB. Phylogenetic analysis revealed that the six marker genes were highly conserved and could be used as the molecular marker for identifying the parents of the distant hybrids in cyprinid fish. The expression patterns of the *scpp5* gene was examined in various tissues, including the brain, gill, heart, liver, muscle, skin, fins, gonad, eye, and kidney, showing that the *scpp5* gene was ubiquitously expressed, indicating its important role in cyprinid fish.

## Introduction

Pharyngeal teeth are not uncommon in Actinopterygii and are histogenetically similar to oral teeth in vertebrates (Sire and Huysseune, 2003). Vertebrate teeth show great diversity in size, shape, structure, and position and play a pivotal role in tracing the evolutionary history of vertebrates (Janvier, 1996; Ungar, 2010). Therefore, the evolutionary origin of teeth has itself been at the center of evolution and development research (Huysseune et al., 2022). In contrast to mammals, Actinopterygii teeth are widely distributed in the mouth and pharynx. However, Cypriniformes lost from the entire mouth cavity and upper pharynx, and only the pharyngeal teeth were reserved on the fifth ceratobranchial bones of the lower posterior pharynx (Aigler et al., 2014), especially the pharyngeal teeth in Cyprinidae exhibit species-specific numbers, morphology, and arrangements, which can be used as an important taxonomical characteristic for Cyprinidae. The morphology of the pharyngeal bone and teeth, as well as the numbers of tooth rows and teeth in each row, is used as one of the taxonomic characters for some genera of Cyprinidae (Chu, 1935; Banarescu, 1967; Krupp and Schneider, 1989). Consequently, identifying the developmental and variation genetic basis of pharyngeal teeth in model species zebrafish becomes the hotspot (Borday-Birraux et al., 2006). Previous studies showed that several signaling pathways played a key role in the position, shape, number, and arrangement of pharyngeal teeth in zebrafish, including the hedgehog signaling, fibroblast growth factor and retinoic acid signaling, ectodysplasin A (EDA)—EDA receptor signaling, and bone morphogenetic protein signaling (Stock, 2007; Aigler et al., 2014; Gibert et al., 2019). The specific expression of secretory calcium-binding phosphoprotein 5 (*scpp5*) gene and ferrintin, heavy polypeptide 1b (*fth1b*) gene in zebrafish pharyngeal teeth development was detected by whole-embryo *in situ* hybridization (Kawasaki et al., 2021; Qu et al., 2021; Zhou et al., 2021). Furthermore, the gene *scpp5* showed a higher expression in the lower pharyngeal jaws of large-toothed cichlid fish than those of the small-toothed ones (Karagic et al., 2020), suggesting that this gene might play a key role in pharyngeal teeth formation. Also, *scpp5* was found to show the highest expression in teeth of zebrafish among all SCPP genes (Kawasaki et al., 2021). A previous study showed that members of the Distal-less-related (Dlx) family of homeodomain transcription factors were expressed at numerous stages of tooth development in the zebrafish and the mouse (Jackman and Stock, 2006), especially, the six dlx (2a, 2b, 3a, 4a, 4b, and 5a) genes underwent extensive diversification of expression of individual genes both within and between dentitions, and they expressed overlaps in time and space, particularly during early morphogenesis (Borday-Birraux et al., 2006). The members of the Dlx family are likely downstream of the cause of oral tooth loss in Cypriniformes (Stock et al., 2006), and they have been used as a good model for the fate of developmental pathways once an initiation signal has been modified in evolution (Aigler et al., 2014; Gibert et al., 2019), especially for the three genes *dlx2b*, *dlx4b*, and *dlx5a*, which expressed more intensively in the dental epithelium (Borday-Birraux et al., 2006; Jackman and Stock, 2006; Renz et al., 2011), and therefore are easily observable. The paired-related homeodomain transcription factor *pitx2* is considered the earliest morphologically visible sign of tooth development (Stock et al., 2006), and the *pitx2* expression is the earliest indicator of zebrafish tooth development (Jackman et al., 2004). *Pitx2* is strongly expressed in bilateral patches of the pharyngeal epithelium joined by the weak expression across the midline beginning at 36 h post-fertilization (hpf), which is much earlier than other initial marker gene expression beginning after 48 hpf (Jackman et al., 2004). These previous studies provide great valuable tools for the in-depth understanding of pharyngeal teeth development in cyprinid fish.

**FIGURE 1:**
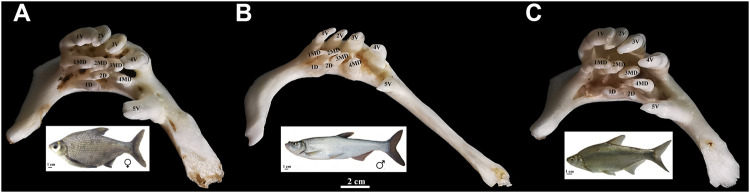
Appearance of pharyngeal teeth in BSB **(A)**, TC **(B)**, and BT **(C)**. Five ventral teeth (positions 1–5V), four mediodorsal teeth (1–4MD), and two dorsal teeth (1D and 2D) in each pharyngeal teeth is shown; bar = 2 cm. The appearance of BSB, TC, and BT are below each of the pharyngeal teeth; bar = 1 cm.

*Megalobrama amblycephala* (blunt snout bream, BSB) and *Culter alburnus* (topmouth culter, TC) are economically important freshwater fish in China (Chen, 1998; Zhou et al., 2008). The hybrid F_1_ (BT) of intergeneric crosses of BSB (♀) × TC (♂) showed many physiological advantages over their parents, such as faster growth rates, higher hypoxia tolerance, and greater disease resistance (Xiao et al., 2014; Li et al., 2018). Also, it is becoming an important freshwater product in Chinese aquaculture. In the cross combination, BSB and TC are herbivorous and carnivorous, respectively, while the BT hybrids have the same feeding habit (herbivorous) as BSB. BT and BSB have almost the same dental formula and shape, number, and arrangement of pharyngeal teeth (Figure 1), but they are different from that of TC. The dietary habits of the hybrid BT were mainly inherited from herbivorous BSB (Xiao, 2013). Until now, the hybrid lineage of BSB (♀) × TC (♂) (F_1_–F_6_) has been established in our fish breeding center, providing a new germplasm resource for fish genetic breeding and the studies of genetics and developmental biology in fish hybridization (Liu et al., 2022). Furthermore, the embryo of BT is similar to that of their parents with a very few pigment and almost transparent, supplying valuable materials for the investigation on the early developmental of pharyngeal teeth in cyprinid fish. Pharyngeal teeth are one of the important feeding organs in cyprinid fish (Zeng and Liu, 2011; Jawad et al., 2022), and the development of pharyngeal teeth likely plays a key role in initial feeding of larvae. However, the time of initial feeding of larvae in aquaculture mainly relies on the traditional experience. It is crucial to figure out the spatiotemporal characteristics of the pharyngeal teeth development and the critical period when the transition occurred from an endogenous to an exogenous nutrition for prelarvae, contributing to increase their survival rates.

In the present study, six genes (*dlx2b*, *dlx4b*, *dlx5a, scpp5*, *pitx2*, and *fth1b*) were used to verify their expression pattern in BT by *in situ* hybridization (ISH) and further investigate early development of pharyngeal teeth in BT and BSB. Furthermore, we analyze the conservation of these genes using the methods of molecular phylogenetics.

## Materials and methods

### Animals

All experiments were approved by the Animal Care Committee of Hunan Normal University and followed guidelines of the Administration of Affairs Concerning Animal Experimentation of China. The embryos of BSB and F_1_ hybrids (BT) of BSB (♀) × TC (♂) were obtained from artificial hybridization in our laboratory (Changsha, Liu) and maintained at 22°C under standard conditions referenced to the zebrafish (Westerfield, 2007). Fifty individuals per sample used for whole-mount ISH were incubated in 1-phenyl-2-thiourea (0.003%) to prevent pigmentation. A series of embryos was fixed at intervals of 4 h from 42 to 216 hpf for whole-mount ISH, and the control specimens from the same ontogenetic phases were immersed in a mixture of glutaraldehyde–paraformaldehyde. Furthermore, the larvae of red crucian carp (HJ), allotetraploid hybrids (4nAT_JL) of red crucian carp × common carp, F_1_ hybrids (JF) of HJ (♀) × BSB (♂), and the F_1_ hybrids (BF) of TC (♀) × BSB (♂) were also sampled from our laboratory. The appearance of pharyngeal teeth of adult BT, BSB, and TC is shown in Figure 1.

### Whole-mount *in situ* hybridization

The whole-mount ISH followed Verstraeten et al. (2012) with a few modifications to increase probe penetration in the pharyngeal region. For each stage, the embryos were fixed overnight at 4°C in a phosphate-buffered saline (PBS) solution containing 4% paraformaldehyde (PFA) and stored at −20°C in methanol. Prelarvae were cleared in 80%–100% glycerol for whole-mount observation and pretreated with 25 μg/ml proteinase K for 30–60 min at room temperature. The hybridization was carried out overnight at 60°C in the solution described by Rosa et al. (2021). Sense and anti-sense RNA probes were generated from 1 μg of linearized PCR2.1-TOPO plasmid using T7 polymerases and then labeled with digoxigenin-dUTP (DIG RNA labeling kit, Roche Diagnostics, Mannheim, Germany). The integrity riboprobes were assessed through agarose gel electrophoresis. The excess probe was removed with four 1-h washes at 60°C in hybridization solution. Specimens were incubated with the anti-digoxigenin-alkaline phosphate antibody overnight at 4°C. The antibody was removed with five 1-h washes at room temperature, followed by an additional wash overnight at 4°C. Probe–antibody complexes were detected by incubation with the BM Purple substrate (Roche) at room temperature for 6–48 h. After hybridization, the embryos were post-fixed in 4% PFA. Some of them were cleared in glycerol and photographed using a stereo microscope (Leica MZ 16FA, Switzerland).

### Probes

We used anti-sense RNA digoxigenin-labeled probes transcribed from BSB and TC cDNA fragments as follows: *dlx2b* (ON734149 and ON734150), *dlx4b* (ON734182 and ON734183), *dlx5a* (ON734215 and ON734216), *pitx2* (ON734259 and ON734260), *fth1b* (ON734291 and ON734292), and *scpp5* (ON734327 and ON734328). Primers for the six probes were examined in different species of cyprinid fish and hybrids in our laboratory, including BSB, TC, BT, BF, HJ, 4nAT_JL, and JF. Primer pairs and PCR conditions are given in Table 1.

**TABLE 1:** Primers used in this study and PCR conditions.

Locus	Primer name	Primer sequence	Source	PCR condition
*scpp5*	Scpp5 F	GAA​ATC​ATT​TTT​CCA​CCG​AGA​TTC​C	ON734327 and ON734328	94°C-5min, (94°C-30s, 60.8°C-30s, and 72°C-24s) 35 cycles,72°C-10min
	Scpp5 R	ATG​TGC​CTG​TCC​TGT​CTG​AGC​CTG		
*fth1b*	fth1bF1	TCT​CAG​GTG​AGG​CAG​AAC​TTC​CAT​C	ON734291 and ON734292	94°C-5min, (94°C-30s, 58.0°C-30s, and 72°C-36s) 35 cycles,72°C-10min
	fth1bR1	TTC​CTT​TCC​CAG​AGT​GTG​CTT​GTC​G		
*pitx2*	Pitx2 F2	TGG​ACC​ACC​ATC​ACC​ACC​ACA​ATC​A	ON734259 and ON734260	94°C-5min, (94°C-30s, 60.1°C-30s, and 72°C-54s) 35 cycles,72°C-10min
	Pitx2 R2	TTC​TGC​ACG​CTC​GCG​TAT​CCA​AAA​C		
*dlx2b*	dlx2bF2	CTT​CAG​TAC​CGT​TCA​CAA​GTC​GCA​G	ON734149 and ON734150	94°C-5min, (94°C-30s, 60.9°C-30s, and 72°C-24s) 35 cycles,72°C-10min
	dlx2bR2	CTG​AAA​ACT​GGA​GTA​GAT​GGT​TCG​C		
*dlx4b*	dlx4bF4	AGT​ACA​TGG​ATT​GCA​CTC​AGG​CGG​A	ON734182 and ON734183	94°C-5min, (94°C-30s, 63.1°C-30s, and 72°C-42s) 35 cycles,72°C-10min
	dlx4bR4	ACA​TCA​TCT​GAG​GTC​TGG​GCA​GAG​G		
*dlx5a*	dlx5aF1	GAG​TAT​TCG​ACA​GAA​GGA​TTC​CGA​G	ON734215 and ON734216	94°C-5min, (94°C-30s, 59.6°C-30s, and 72°C-24s) 35 cycles,72°C-10min
	dlx5aR1	CCG​TTG​ACC​ATC​CTC​ACT​TCG		

### Cloning and sequence analysis

RNA was isolated from larvae of BSB, TC, BT, BF, HJ, 4nAT_JL, and JF using TRIzol reagent (Invitrogen, California, CA, United States) as described in the manufacturer’s instruction. The first-strand cDNA was synthesized using the Revert Aid First Strand Synthesis Kit (Thermo Fisher Scientific, Waltham, MA, United States). The synthesis of six genes was carried out using the specific primers in Table 1, and the PCR products were cloned into the pMD18-T vector (TaKaRa, Dalian, China) and subjected to automated sequencing. All sequences used in this study have been deposited in the GenBank database (GenBank Accession Numbers ON734151–ON734342). The obtained sequences were screened using BLAST searches of GenBank, and the ClustalW (http://www.ebi.ac.uk/) and MEGA 11.0 programs were used to determine identity. Of the clones sequenced, six were determined by phylogenetic analyses to represent each gene.

### Quantitative real-time PCR

Total RNA was extracted from different tissues (pharyngeal teeth, brain, gill, heart, liver, muscle, skin, fins, gonad, eye, and kidney) of BT and its parents (BSB and TC), including the 7-month-old juvenile and 2-year-old adult fish. The high specificity marker *scpp5* gene was further used to investigate the gene expression pattern. The qPCR primer of *scpp5* gene was synthesized by Shanghai Sangon Company (Shanghai, China). Amplification and detection of the fluorescence were measured using a 7500 Real-Time PCR System (Applied Biosystems, Foster City, CA, United States) and the PowerUp SYBR Green Master Mix (Thermo Fisher Scientific). The *β-actin* gene was used as an internal positive control. qPCR was carried out in a 10 µl mixture containing 1 µl RNA, 5 µl biomarker 2 × SYBR Green Fast qPCR Mix (Thermo Fisher Scientific), 0.50 µmol/L forward primer, 0.50 µmol/L reverse primer, and 0.20 µmol/L of ROX reference dye. Forty cycles of amplification (95°C for 15 s, 60°C for 2 min, and 72°C for 15 s) were performed after denaturing at 95°C for 10 min and 50°C for 2 min. The expression level of *scpp5* was analyzed using the comparative threshold cycle method (2^−ΔΔCT^) with β-actin (ACTB) as an internal reference. PCR efficiency (E) and correlation coefficient (R^2^) were conducted as previously described (Fleige et al., 2006). The experiment was performed three times and each time with three fish. The comparison between the expression of eye and other tissues was performed using the one-way analysis of variance (ANOVA) statistical method using STATISTICA 10.0 (StatSoft), *p*-values of less than 0.05 were considered statistically significant.

### Phylogenetic analysis

Three to four individuals of each species were used in phylogenetic analyses. MAFFT in PhyloSuite v1.2.2 (Zhang et al., 2020) was used to align all the sequences, as well as the related genes of other cyprinid fish and non-cyprinid fishes in GenBank (Supplementary Table S1). Three species from Perciformes and Scorpaeniformes, including *Betta splendens*, *Anabas testudineus*, and *Cyclopterus lumpus*, were used to represent outgroup (Supplementary Table S1) in the phylogenetic analysis. We tested the homogeneity of the combined genes (*dlx2b* + *dlx4b* + *dlx5a* + *pitx2* + *fth1b* + *scpp5*) in *PAUP, and the *p*-value was 0.054 (>0.05). Consequently, the aligned six nucleotide sequences were concatenated to construct the phylogenetic tree using the maximum likelihood (ML) method implemented in RAxML v8.2.4 (Stamatakis, 2014) with 1,000 bootstrap replicates. Each gene was treated as unlinked, since it is not required to have the same individuals analyzed for every genetic marker if partitions are considered unlinked (Sota and Vogler, 2001; Husemann et al., 2013). Bayesian inference (BI) analysis was also conducted in MrBayes v3.2.6 (Ronquist et al., 2012) using the Markov chain Monte Carlo method with 10 million generations and sampling trees every 1,000 generations. The analysis was terminated after the average standard deviation of the split frequencies fell to less than 0.01. The first 25% of trees were discarded as burn-in with the remaining trees being used for generating a consensus tree. The final trees were visualized in FigTree v1.4.4 (Rambaut, 2018).

## Results

### Probe synthesis

The primers of six probes were successfully designed according to the BSB and TC genomic library (Ren et al., 2019) and amplified in BT, BSB, TC, HJ, 4nAT_JL, and JF (Supplementary Figure S1, Table 1). The PCR products with the cDNA template of BT and BSB were checked by sequencing and subsequently used to synthesize the probe.

### Whole-mount *in situ* hybridization

**FIGURE 2:**
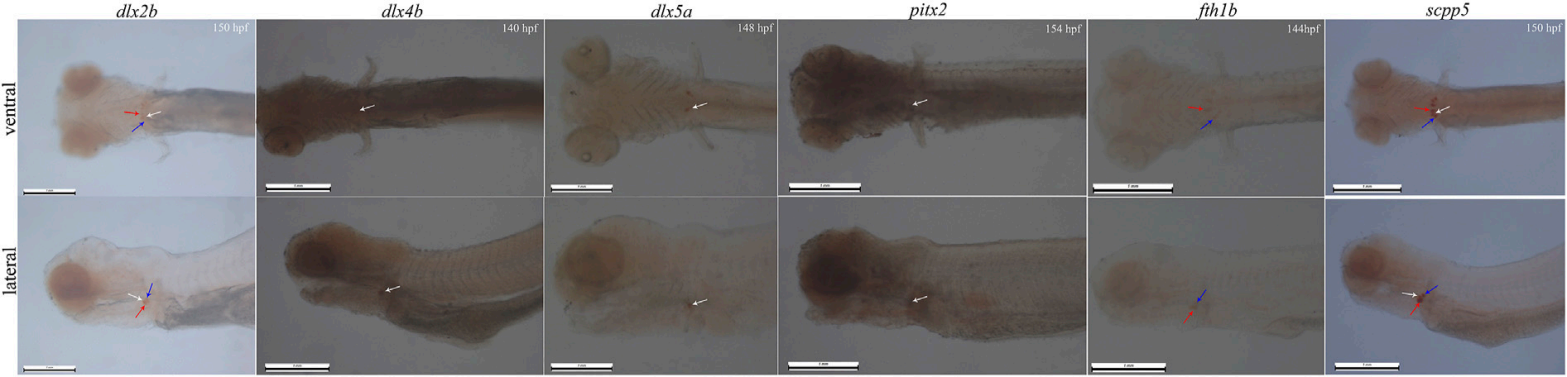
Whole-mount *in situ* hybridization for six genes (*dlx2b*, *dlx4b*, *dlx5a*, *pitx2, fth1b*, and *scpp5*) in BT. Pharyngeal region. Anterior is to the left. Ventral (upper) and lateral (lower) views showing tooth location during 140–154 h post-fertilization (hpf) prelarval hybridized for the six genes. The 140–154 hpf was determined by the previous investigation on the expression of the gene *scpp5* occurred in 4V^1^, 3V^1^, and 5V^1^. The white arrow refers to 4V, the red arrow refers to 3V, and the blue arrow refers to 5V. 4V^1^, 3V^1^, and 5V^1^ strongly express *dlx2b* and *scpp5*, while only 4V^1^ weakly expresses *dlx4b* and *pitx2*; *dlx5a* is only expressed in the early developing 4V^1^, and *fth1b* is expressed in the early developing 3V^1^ and 5V^1^. Scale bar = 1 mm.

In whole-mount-hybridized prelarvae, teeth showing a signal (Figure 2) can readily be identified by referring to the timetable of development (140–154 hpf). By ventral view, developing teeth could be seen in symmetrical loci, at both sides and posterior to the fifth branchial arch. By lateral view, developing teeth are identified as round spots located posterior to the fifth branchial arch.

In whole-mount-hybridized prelarvae, the six genes were expressed in different locations within the BT dentition, and the difference in specificity (Figure 2) was observed. *Dlx2b* expression was observed during the development of the teeth 4V^1^, 3V^1^, and 5V^1^ with a little strong signal, while *scpp5* expression was observed on 4V^1^, 3V^1^, and 5V^1^ with a very strong signal. The expression patterns of *dlx4b* and *pitx2* were difficult to assess in the pharyngeal region as a strong masked signal. The *dlx5a* expression was activated exclusively in the developing 4V^1^, but not in the developing 3V^1^ and 5V^1^. In contrast, the *fth1b* expression was observed during the development of 3V^1^ and 5V^1^ but not at 4V^1^.

**FIGURE 3:**
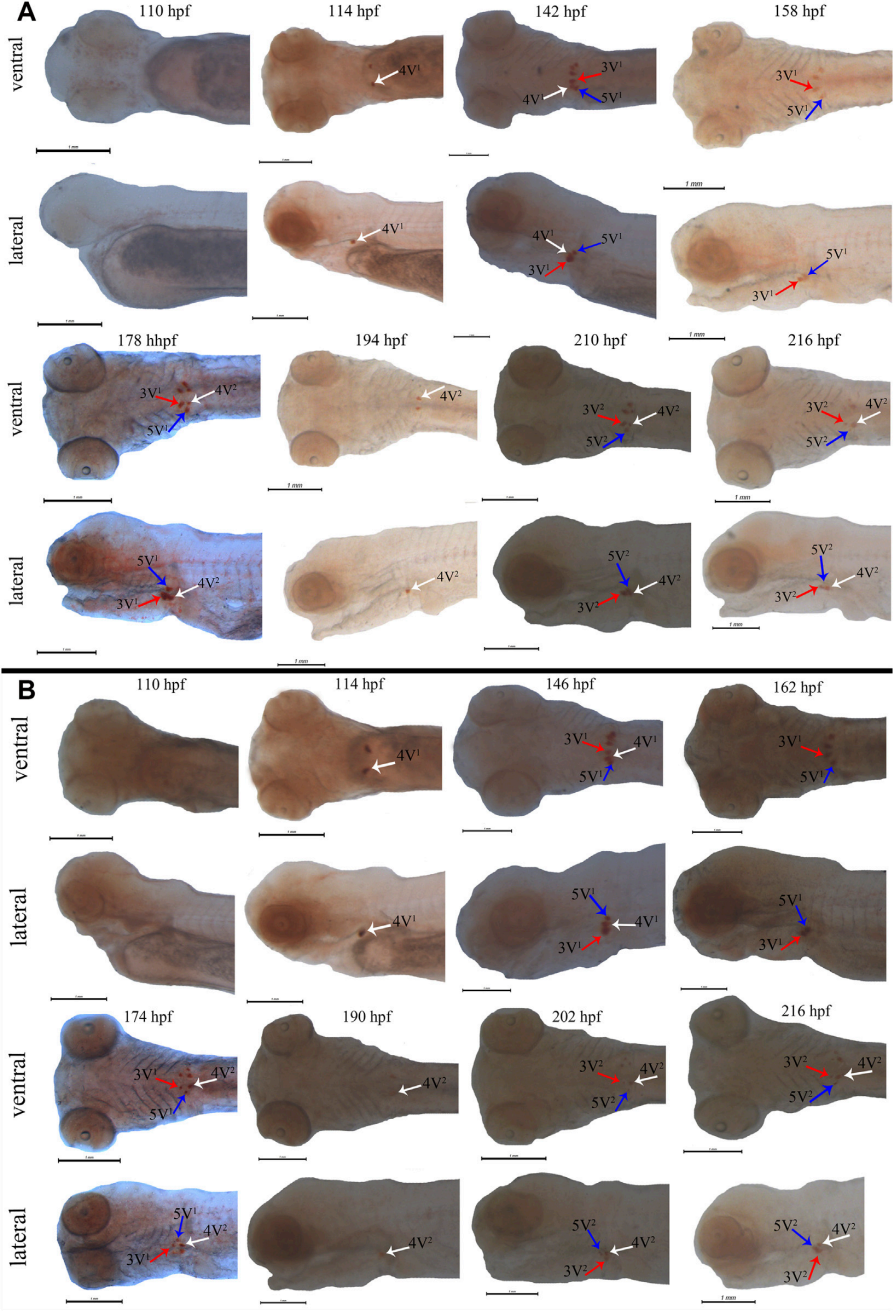
Whole-mount *in situ* hybridization for *scpp5* in BT **(A)** and BSB **(B)**. At 114 hpf, 4V^1^ activates *scpp5* expression both in BT and BSB, and 3V^1^ and 5V^1^ strongly express *scpp5* at 142 hpf in BT and 146 hpf in BSB. At 158 hpf and 162 hpf, the expression of *scpp5* in 4V^1^ disappears in BT and BSB, respectively, and 4V^2^ expresses *scpp5* at 178 hpf (BT) and 174 hpf (BSB). The absence of *scpp5* expression in 3V^1^ and 5V^1^ occurs at 194 hpf (BT) and 190 hpf (BSB), and 3V^2^ and 5V^2^ express *scpp5* at 210 hpf (BT) and 202 hpf (BSB), respectively. Scale bar = 1 mm.

Subsequently, the *scpp5* gene was further used to investigate the spatiotemporal characteristics of pharyngeal teeth in BT and BSB. At 114 hpf, *scpp5* transcription was activated in 4V^1^, and the signal remained strong until 142 hpf in BT while 146 hpf in BSB. Meanwhile, 3V^1^ and 5V^1^ started to express this gene. At 158 hpf and 162 hpf, respectively, *scpp5* expression in 4V^1^ was difficult to be observed in whole-mount of BT and BSB, while 3V^1^ and 5V^1^ were still strongly labeled until 178 hpf and 174 hpf, respectively. In larvae older than this time, 4V^2^ in BT and BSB started to express *scpp5* until 216 hpf with the weaker signal but still visible (Figures 3A,B). The absence of expression in 3V^1^ and 5V^1^ was both found at 194 hpf in BT and at 190 hpf in BSB. Then, the developing 3V^2^ and 5V^2^ started to express *scpp5* in BT (210 hpf) and BSB (202 hpf) and remained labeled until 216 hpf both in BT and BSB (Figures 3A,B). In larvae older than 216 hpf, the *scpp5* expression was still observed but with a very obscure signal, thus making it difficult to distinguish between 4V, 3V, or 5V.

### Tissue expression of *scpp5* gene in BT, blunt snout bream, and topmouth culter

**FIGURE 4:**
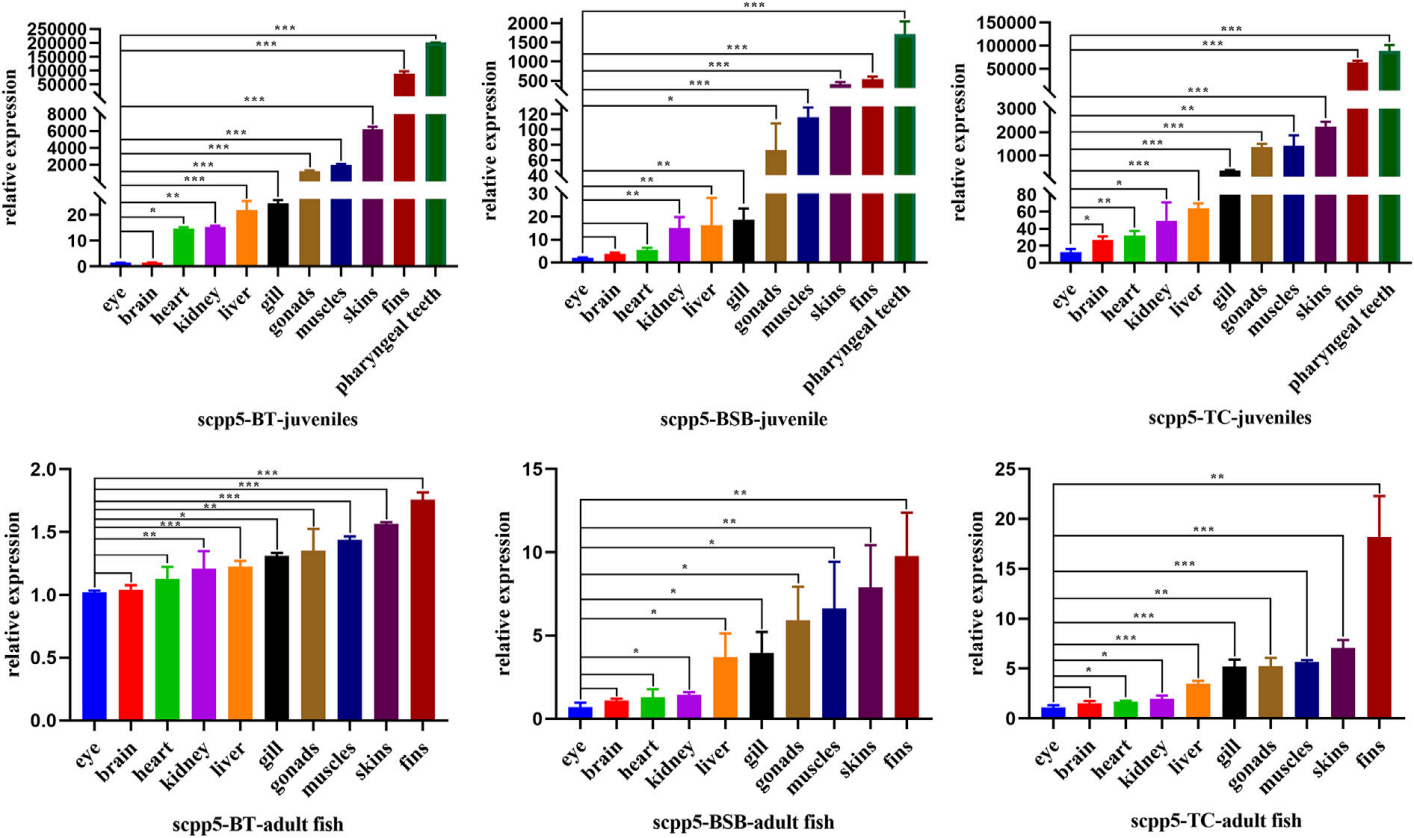
*Scpp5* expression in pharyngeal teeth, brain, gill, heart, liver, muscle, skin, fins, gonad, eye, and kidney of BT, BSB, and TC were determined by quantitative real time PCR. Data are plotted as mean ± SE of three independent experiments. The β-actin gene is used as an internal positive control. Evaluation of the variation between the results was performed using the one-way ANOVA test. * means 0.01 < *p* < 0.05, ** means 0.001 < *p* < 0.01, and *** means *p* < 0.001.

*Scpp5* gene exhibited high specificity during the development of pharyngeal teeth in BT and BSB. We further analyzed the expression of *scpp5* in BT and its parents using quantitative real-time PCR (Figure 4). We observed that most of the *scpp5* gene was widely expressed in all investigated tissues with very different expression levels. The similar relative expression patterns among BT, BSB, and TC were detected in juvenile (7 months old) and adult fish (2 years old). The highest expression level of *scpp5* in the pharyngeal teeth and the lowest in eyes were found in juveniles. As the pharyngeal teeth in adult fish were so hard to extract RNA, the highest expression was found in fins and the lowest in eye. However, the evaluation of the variation between eye and other tissues was not exactly the same in the juvenile or adult of the three types of fish (Figure 4). The relative expression in the eye was extremely significantly different (*p* < 0.001) from that in skin, fins, and pharyngeal teeth in the juvenile of all three types of fish. There is no significant difference (*p* > 0.05) between the eye and brain in adult fish of all three types of fish.

### Phylogenetic analysis

**FIGURE 5:**
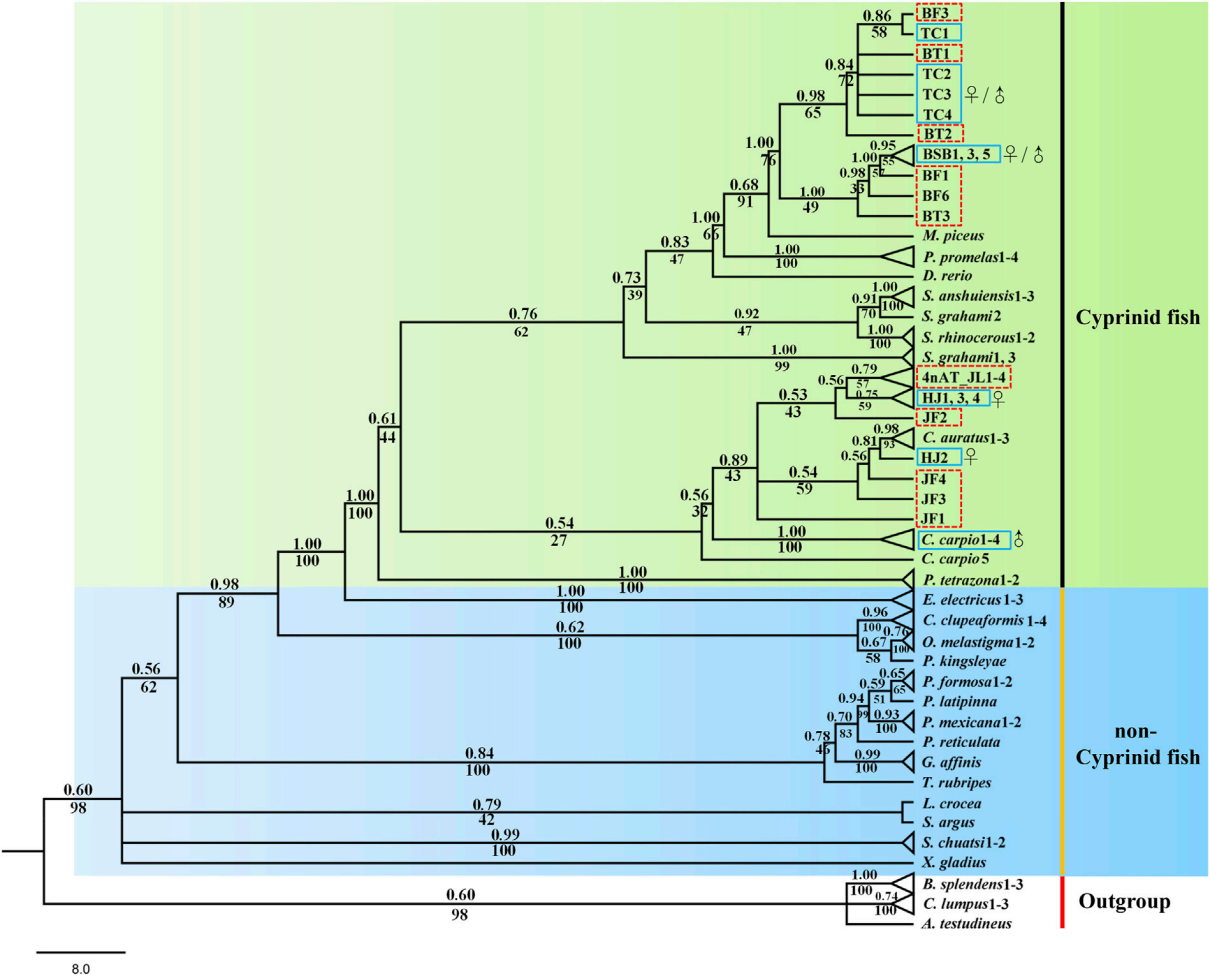
Phylogenetic tree based on Bayesian inference using the concatenate sequences (*dlx2b* + *dlx4b* + *dlx5a* + *pitx2* + *fth1b* + *scpp5*); values on branches indicate Bayesian posterior probabilities and bootstrap proportions from a maximum likelihood analysis. The blue solid line means the parents, and the red dotted line means the distant hybrids in the present study. ♀ means female parent, and ♂ means male parent.

Both phylogenetic trees (ML and BI) showed a very similar and robust topology. The phylogenetic analysis revealed that the combined six genes showed a clear identification of the species of cyprinid fish and other non-cyprinid fish (Figure 5), indicating that all the six genes are highly conserved in Actinopterygii. Most of the individuals of the same species formed a monophyletic clade with high support values, and the species from the same genus showed a closer relationship, especially the phylogenetic topologies provided a clear parent–child relationship for the distant hybrids, and the hybrids nested with their parents. For instance, a chimeric structure was found in the phylogenetic tree between BT and its parents (BSB and TC), as well as between 4nAT_JL and its parents (red crucian carp and common carp), and between JF and its parents (HJ and BSB). Furthermore, the distant hybrids have a closer relationship with the female parent in most cases.

## Discussion

ISH plays an important role in tracing early development of pharyngeal teeth in cyprinid fish. Several signal paths control the development of pharyngeal teeth, and many genes have a specific expression during this process, such as the Dlx gene family and FGF gene family (Borday-Birraux et al., 2006; Stock et al., 2006; Aigler et al., 2014; Gibert et al., 2019), especially the high specific expression of *scpp5* was found in zebrafish recently (Kawasaki et al., 2021; Qu et al., 2021). The whole-mount ISH can visualize the formation of teeth, contributing to seize the crucial period of transformation of nutrient source in the prelarval stage. Hence, the technology of ISH used in the development of pharyngeal teeth plays a potential application value on the breeding of cyprinid fish. Pharyngeal teeth is an important feeding organ in cyprinid fish (Zeng and Liu, 2011; Jawad et al., 2022), while there is no study available about the role of development of pharyngeal teeth when the prelarval translating into external feeding. Our preliminary results showed that the *scpp5* expression of first 4V^1^ in BT and BSB emerged at 114 hpf (Figure 3) and subsequently mineralized at 154 hpf (data not shown), while *Artemia salina*, often used for feeding individuals in the prelarval stage, was just appeared in the enteric canal of prelarvae at 166 hpf (data not shown), suggesting that the prelarvae initiated feeding on external nutrition after teeth mineralization. Also, further studies should be executed to verify the role of teeth mineralization on feeding on exogenous nutrient in prelarvae. We successfully designed six RNA probes and used in whole-mount ISH of BT according to the marker genes (*dlx2b*, *dlx4b*, *dlx5a*, *scpp5*, *pitx2*, and *fth1b*) for the development of teeth in zebrafish. Our results showed that all the six genes were expressed in the teeth-developing region (Figure 2), especially for gene *scpp5* with highly specific and strongly expressed in tooth-specific loci 4V^1^, 5V^1^, and 3V^1^ (Figure 2). Furthermore, *scpp5* gene was expressed in different tissues in juveniles and adults of BT, BSB, and TC, with various expression levels (Figure 4).

There are eight genes in the Dlx family, in which six genes (*dlx2a*, *dlx2b*, *dlx3b*, *dlx4a*, *dlx4b*, and *dlx5a*) are expressed during the pharyngeal dentition development in zebrafish (Borday-Birraux et al., 2006). The expression of the six zebrafish Dlx genes overlaps in time and space, particularly during early morphogenesis, and teeth in different locations and generations within the zebrafish dentition differ in the number of genes expressed. However, only three genes (*dlx2b*, *dlx4b*, and *dlx5a*) were expressed during the development of pharyngeal teeth in BT, with a little different expression pattern from that in zebrafish. Two genes *dlx2b* and *dlx5a* were both expressed at 4V^1^, 3V^1^, and 5V^1^ in zebrafish (Borday-Birraux et al., 2006), while only *dlx2b* was expressed at the three loci and *dlx5a* was just expressed at 4V^1^ in BT (Figure 2). Furthermore, a previous study showed a clear *dlx4b* expression in all developing phases of 4V, 3V, and 5V in zebrafish (Borday-Birraux et al., 2006); however, *dlx4b* was difficult to assess because of a strong labeling in the pharyngeal region and to estimate the concrete position of 4V, 3V, or 5V in BT (Figure 2).

Ferritin genes are expressed in various cells in many kinds of animals (Arosio et al., 2015). The expression pattern of *fth1b* was similar to that of the known zebrafish pharyngeal teeth marker *dlx2b* and was specifically expressed in the zebrafish pharyngeal teeth during development but mainly expressed at 3V^1^ and 5V^1^ (Zhou et al., 2021). The same result of the specific expression of *fth1b* was found at 3V^1^ and 5V^1^ of BT (Figure 2). The *fth1b* gene had a strong labeling in the pharyngeal region while without any labeling in other areas of the BT whole-mount ISH. It also can be used as a specific gene marker for pharyngeal teeth during development.

The early developmental pattern of pharyngeal teeth in BT and BSB coincided with that in zebrafish according to the whole-mount ISH (Gibert et al., 2019). They have very similar dental formula (2·4·5–5·4·2) (Figure 1), which were arranged in three distinct tooth rows, a ventral (V), a mediodorsal (MD), and a dorsal (D) in adults, having five (positions 1–5V), four (1–4MD), and two (1D and 2D) teeth, respectively (Van der Heyden and Huysseune, 2000; Wautier et al., 2001). The first emergence tooth was found on the ventral rows, named 4V^1^, and the formation of the subsequent teeth 3V^1^ and 5V^1^ was almost at the same time (Figure 3). The row 4V^1^ was verified to serve as an initiator controlling the formation of the subsequent teeth in zebrafish (Gibert et al., 2019). However, the emergence time of 4V^1^ in BT and BSB (114 hpf) was far post-dated that in zebrafish (48 hpf), due to the very fast embryo growth in zebrafish with higher incubation temperature (>28°C). Furthermore, the *scpp5* transcription was activated in 4V^1^ almost at the same time in BT and BSB, while the expression in 3V^1^ and 5V^1^ and the absence in 4V^1^ in BT (142 hpf and 158 hpf, Figure 3A) occurred earlier than those in BSB (146 hpf and 162 hpf, Figure 3B). However, the developing 4V^2^, 3V^2^, and 5V^2^ started to express *scpp5* in BSB at 174 hpf and 202 hpf (Figure 3B), which were earlier than that in BT (178 hpf and 210 hpf, Figure 3A), suggesting that the rate of pharyngeal tooth replacement was faster in BSB than that in BT. In addition, with the development and the growth of the pharyngeal bone, the relative positions of 4V^2^, 3V^2^, and 5V^2^ changed compared with those of 4V^1^, 3V^1^, and 5V^1^ (Figure 3).

Kawasaki (2009) reported the repertoire of SCPPs in zebrafish and their expression in dental and skeletal tissues. The family of secretory calcium-binding phosphoproteins includes SCPPs involved in bone and dentin formation, as well as proteins involved in enamel formation, milk caseins, and some salivary proteins, while only *scpp5* was verified as a good starting point to elucidate the nature of the “bone of attachment” in the zebrafish dentition (Qu et al., 2021; Rosa et al., 2021). Highly similar to the expression pattern of *scpp5* in zebrafish, there were strong labeling of *scpp5* at 4V, 3V, and 5V in BT (Figure 3), and our results showed that this gene was exclusively expressed at the developing pharyngeal teeth. Therefore, a further study on the expression pattern of *scpp5* was performed on juvenile and adult fish of BT, BSB, and TC. The *scpp5* gene was widely expressed in all investigated tissues, suggesting their universal functions in teleost (Karagic et al., 2020), especially in cyprinid fish. In cichlid fish, *scpp5* had a higher expression in the lower pharyngeal jaws of large-toothed species than those of the small-toothed ones (Karagic et al., 2020), and even the pharyngeal teeth in the scpp5^−/−^ zebrafish exhibited a decrease in numbers of functional teeth (Qu et al., 2021), suggesting that this gene plays a key role in pharyngeal teeth formation. Our results showed that there were the highest expression levels of *scpp5* at pharyngeal teeth in juvenile tissues. *Scpp5* is found only in actinopterygians and expressed during the formation of hypermineralized tissues on scales and teeth (Kawasaki et al., 2021). Also, various SCPP genes are expressed in the skin and jaw during the formation of bone, teeth, and scales in osteichthyans (Mikami et al., 2022). The bone is widely found in the dermal skeleton and the endoskeleton, and dentin is present in teeth, scales, fin rays, and other dermal skeletal units (Donoghue et al., 2006). The surface of teeth and dermal skeletal units is often covered with hypermineralized tissues (Schultze, 2016), which can be detected using *scpp5* gene expression. The highest expressions of *scpp5* gene at pharyngeal teeth in juvenile fish (7 months old) and at fin rays in adult fish (2 years old) were found in the present study, consistent with the variational trend in BT, BSB, and TC. Our results indicated a higher expression at fin rays, skin, and muscle in juvenile fish, but a low level and relatively stable expression in adult fish (Figure 4). All of the aforementioned results suggest that the *scpp5* can be used as an excellent gene marker for investigating the development of the skeletal system in actinopterygians.

Phylogenetic analyses can provide us with strong evidence for correctly naming genes and analyze the conservation of genes (Jiang et al., 2016). In total, six genes were mainly used to explore the tooth developmental biology in previous studies (Borday-Birraux et al., 2006; Jackman and Stock, 2006; Aigler et al., 2014; Gibert et al., 2019), and several studies used the phylogenetic tree to identify the accuracy of these genes (Jackman and Stock, 2006; Stock, 2007; Lv et al., 2017; Kawasaki et al., 2021). In the present study, the designed primers for the six genes are universal and can be amplified in different cyprinid fish and their distant hybrids (Figure 5). Our results showed that the six genes has high conservatism in cyprinid fish, exhibiting good species recognition. Furthermore, the distant hybrids nested in their parents in the phylogenetic tree. For example, 4nAT_JL formed a single genetic cluster, which was located between the red crucian carp (HJ) and common carp (Figure 5). The similar situation was found in the distant hybrids BT, as well as the distant hybrids JF. Consequently, we found not only satisfactory evidence of interspecies phylogenetic relationships among cyprinid fish but also the nested relationship between distant hybrids and their parents using the six initiation marker genes for pharyngeal teeth. Previous studies suggested that interspecies hybridization is not uncommon in fish, including the families Poeciliidae, Atherinidae, Cyprinidae, and Cobitidae (Gu et al., 2022). Our phylogenetic tree with these genes can be used to uncover the parents of distant hybrids, which is also of significance in application of identifying the hybrids and their parents.

## Conclusion

Overall, we selected six marker genes (*dlx2b*, *dlx4b*, *dlx5a*, *pitx2, fth1b*, and *scpp5*) for investigating the development of pharyngeal teeth. Our results showed that there existed differences in the expression of the six genes in the pharyngeal region. Also, a highly specific marker gene *scpp5* playing roles in the early developing pharyngeal teeth was found in the present study. The *scpp5* expression pattern established a clear graphic representation on the spatiotemporal characteristics of the early morphogenesis of 4V^1^, 3V^1^, and 5V^1^, as well as the replacement teeth 4V^2^, 3V^2^, and 5V^2^. The *scpp5* genes were ubiquitously expressed in hybrids BT and its parents, but highly expressed in the tissues that are more likely to be involved with this gene, indicating the critical roles of this gene in fish skeletal system regulation. The phylogenetic tree showed a high conservation of the six marker genes in Actinopterygii, especially the potential key role in identifying the parents of the hybrids in cyprinid fish.

## Data Availability

The datasets presented in this study can be found in online repositories. The names of the repository/repositories and accession number(s) can be found in the article/Supplementary Material.
