# Supplementary material for: Spatiotemporal characteristics of the pharyngeal teeth in interspecific distant hybrids of cyprinid fish: Phylogeny and expression of the initiation marker genes
Source: Front Genet. 2022 Aug 16;13:983444. doi: 10.3389/fgene.2022.983444 (PMC9424816; doi:10.3389/fgene.2022.983444)
Supplement: Supplementary file 1 [file Table1.DOCX]

Table S1 Sequence information included the GenBank accession number, species name and ID for reconstructing the Bayesian Inference (BI) and the maximum likelihood (ML) phylogenetic tree based on 78 individuals from cyprinid fish and non-cyprinid fish, in which 7 individuals were used as outgroup. New sequences for the present article are ON734151–ON734342. Other accession numbers are sequences from the GenBank. NA indicates the data missing.

|  | **ID** | **Taxon** | ***dlx2b*** | ***dlx4b*** | ***dlx5a*** | ***pitx2*** | ***fth1b*** | ***scpp5*** |
| --- | --- | --- | --- | --- | --- | --- | --- | --- |
| Ingroup | BSB1 | *Megalobrama amblycephala* | ON734151 | ON734190 | ON734227 | ON734273 | ON734298 | ON734329 |
|  | BSB3 | *Megalobrama amblycephala* | ON734152 | ON734192 | ON734228 | ON734275 | ON734300 | ON734331 |
|  | BSB5 | *Megalobrama amblycephala* | ON734153 | ON734194 | ON734229 | NA | ON734302 | NA |
|  | TC1 | *Culter alburnus* | ON734176 | ON734211 | ON734240 | ON734288 | ON734323 | NA |
|  | TC2 | *Culter alburnus* | ON734177 | ON734212 | ON734241 | ON734289 | ON734324 | ON734341 |
|  | TC3 | *Culter alburnus* | ON734178 | ON734213 | ON734242 | ON734290 | ON734325 | ON734342 |
|  | TC4 | *Culter alburnus* | ON734179 | ON734214 | ON734243 | NA | ON734326 | NA |
|  | HJ1 | *Carassius auratus* red var. | ON734163 | ON734202 | ON734249 | ON734280 | ON734309 | NA |
|  | HJ3 | *Carassius auratus* red var. | ON734165 | ON734204 | ON734251 | ON734282 | ON734311 | NA |
|  | HJ4 | *Carassius auratus* red var. | ON734166 | ON734205 | ON734252 | ON734283 | ON734312 | NA |
|  | 4nAT_JL1 | *Carassius auratus* red var. (♀) ×  *Cyprinus carpio* (♂) | ON734168 | ON734205 | ON734258 | ON734285 | ON734313 | NA |
|  | 4nAT_JL2 | *Carassius auratus* red var. (♀) ×  *Cyprinus carpio* (♂) | ON734169 | ON734206 | NA | ON734286 | ON734314 | NA |
|  | 4nAT_JL3 | *Carassius auratus* red var. (♀) ×  *Cyprinus carpio* (♂) | ON734170 | ON734207 | NA | ON734287 | ON734315 | NA |
|  | 4nAT_JL4 | *Carassius auratus* red var. (♀) ×  *Cyprinus carpio* (♂) | ON734171 | ON734208 | NA | NA | ON734316 | NA |
|  | BT1 | *Megalobrama amblycephala* (♀) *×*  *Culter alburnus* (♂) | ON734158 | ON734197 | ON734231 | ON734277 | ON734304 | ON734332 |
|  | BT2 | *Megalobrama amblycephala* (♀) *×*  *Culter alburnus* (♂) | ON734159 | ON734198 | ON734232 | ON734278 | NA | ON734333 |
|  | **ID** | **Taxon** | ***dlx2b*** | ***dlx4b*** | ***dlx5a*** | ***pitx2*** | ***fth1b*** | ***scpp5*** |
|  | BT3 | *Megalobrama amblycephala* (♀) *×*  *Culter alburnus* (♂) | ON734160 | ON734199 | ON734233 | ON734279 | ON734305 | ON734334 |
|  | BF1 | *Culter alburnus* (♀) ×  *Megalobrama amblycephala* (♂) | NA | ON734184 | ON734217 | ON734261 | ON734293 | NA |
|  | BF3 | *Culter alburnus* (♀) ×  *Megalobrama amblycephala* (♂) | ON734155 | ON734186 | ON734219 | ON734262 | ON734295 | NA |
|  | BF6 | *Culter alburnus* (♀) ×  *Megalobrama amblycephala* (♂) | ON734156 | ON734189 | ON734222 | ON734266 | NA | NA |
|  | JF1 | *Carassius auratus* red var. (♀) ×  *Megalobrama amblycephala* (♀) | ON734172 | NA | ON734253 | ON734268 | ON734318 | ON734335 |
|  | JF2 | *Carassius auratus* red var. (♀) ×  *Megalobrama amblycephala* (♀) | ON734173 | ON734206 | ON734254 | ON734269 | ON734319 | ON734336 |
|  | JF3 | *Carassius auratus* red var. (♀) ×  *Megalobrama amblycephala* (♀) | ON734174 | ON734207 | ON734255 | ON734270 | ON734320 | ON734337 |
|  | JF4 | *Carassius auratus* red var. (♀) ×  *Megalobrama amblycephala* (♀) | ON734175 | ON734208 | ON734256 | ON734271 | ON734321 | ON734338 |
|  | *C. auratus*1 | *Carassius auratus* | XM_026204367 | XM_026223104 | XM_026231596 | XM_026258711 | NA | XM_026260809 |
|  | *C. auratus*2 | *Carassius auratus* | NA | XM_026277234 | XM_026288781 | XM_026264418 | NA | NA |
|  | *C. auratus*3 | *Carassius auratus* | NA | NA | NA | XM_026225280 | NA | NA |
|  | *C. carpio*1 | *Cyprinus carpio* | XM_019122406 | XM_019110623 | XM_019124617 | XM_019102441 | XM_042753115 | XM_042717614 |
|  | *C. carpio*2 | *Cyprinus carpio* | XM_042753916 | XM_019085282 | XM_019125232 | XM_042770184 | NA | XM_042717615 |
|  | *C. carpio*3 | *Cyprinus carpio* | NA | NA | NA | XM_042770185 | NA | NA |
|  | *C. carpio*4 | *Cyprinus carpio* | NA | NA | NA | XM_042770186 | NA | NA |
|  | *C. carpio*5 | *Cyprinus carpio* | NA | NA | NA | EF051103 | NA | NA |
|  | *M. piceus* | *Mylopharyngodon piceus* | NA | NA | NA | NA | KY926440 | NA |
|  | **ID** | **Taxon** | ***dlx2b*** | ***dlx4b*** | ***dlx5a*** | ***pitx2*** | ***fth1b*** | ***scpp5*** |
|  | *P. promelas* 1 | *Pimephales promelas* | XM_039657433 | XM_039665448 | XM_043253023 | XM_039664504 | NA | XM_039648512 |
|  | *P. promelas* 2 | *Pimephales promelas* | NA | NA | NA | XM_039664505 | NA | NA |
|  | *P. promelas* 3 | *Pimephales promelas* | NA | NA | NA | XM_039664506 | NA | NA |
|  | *P. promelas* 4 | *Pimephales promelas* | NA | NA | NA | XM_039664507 | NA | NA |
|  | *P. tetrazona* 1 | *Puntigrus tetrazona* | XM_043231297 | NA | NA | XM_043256693 | XM_043228002 | XM_043233057 |
|  | *P. tetrazona* 2 | *Puntigrus tetrazona* | XM_043231225 | NA | NA | NA | NA | NA |
|  | *S. anshuiensis* 1 | *Sinocyclocheilus anshuiensis* | XM_016507226 | XM_016440788 | XM_016456639 | XM_016453097 | NA | NA |
|  | *S. anshuiensis* 2 | *Sinocyclocheilus anshuiensis* | XM_016500246 | XM_016455230 | XM_016462710 | XM_016461172 | NA | NA |
|  | *S. anshuiensis* 3 | *Sinocyclocheilus anshuiensis* | NA | XM_016455231 | NA | XM_016255984 | NA | NA |
|  | *S. grahami* 1 | *Sinocyclocheilus grahami* | XM_016295307 | XM_016240727 | XM_016280884 | XM_016255984 | NA | NA |
|  | *S. grahami* 2 | *Sinocyclocheilus grahami* | NA | XM_016240728 | NA | XM_016295130 | NA | NA |
|  | *S. graham* 3 | *Sinocyclocheilus grahami* | NA |  | NA | XM_016295129 | NA | NA |
|  | *S. rhinocerous* 1 | *Sinocyclocheilus rhinocerous* | XM_016527234 | XM_016513264 | XM_016547955 | XM_016552426 | NA | NA |
|  | *S. rhinocerous* 2 | *Sinocyclocheilus rhinocerous* | NA | XM_016513265 | XM_016547648 | XM_016552427 | NA | NA |
|  | *D. rerio* | *Danio rerio* | NM_131297 | BC092714 | BC083280 | AF132446 | NM_001004562 | NM_001145236 |
|  | *E. electricus* 1 | *Electrophorus electricus* | XM_027024826 | XM_027021884 | XM_027012126 | XM_027014181 | NA | XM_035530276 |
|  | *E. electricus* 2 | *Electrophorus electricus* | NA | NA | XM_027012127 | XM_035532216 | NA | NA |
|  | *E. electricus* 3 | *Electrophorus electricus* | NA | NA | XM_027012127 | XM_027014182 | NA | NA |
|  | *G. affinis* 1 | *Gambusia affinis* | NA | NA | NA | XM_044127242 | XM_044124715 | XM_044113462 |
|  | *G. affinis* 2 | *Gambusia affinis* | NA | NA | NA | NA | XM_044124716 | NA |
|  | *O. melastigma* 1 | *Oryzias melastigma* | NA | NA | NA | NA | XM_024282672 | XM_024290127 |
|  | *O. melastigma* 2 | *Oryzias melastigma* | NA | NA | NA | NA | XM_024282673 | NA |
|  | *P. formosa* 1 | *Poecilia formosa* | NA | XM_007558976 | NA | XM_007551061 | XM_007556918 | NA |
|  | *P. formosa* 2 | *Poecilia formosa* | NA | NA | NA | NA | XM_007556919 | NA |
|  | *P. latipinna* | *Poecilia latipinna* | NA | XM_015059994 | NA | XM_015055579 |  | NA |
|  | **ID** | **Taxon** | ***dlx2b*** | ***dlx4b*** | ***dlx5a*** | ***pitx2*** | ***fth1b*** | ***scpp5*** |
|  | *P. mexicana* 1 | *Poecilia mexicana* | NA | NA | NA | XM_014998981 | XM_015012162 | NA |
|  | *P. mexicana* 2 | *Poecilia mexicana* | NA | NA | NA | NA | XM_015012163 | NA |
|  | *P. reticulata* | *Poecilia reticulata* | NA | NA | NA | XM_008421035 | XM_008412099 | NA |
|  | *L. crocea* | *Larimichthys crocea* | NA | XM_010736300 | NA | NA | XM_010743203 | NA |
|  | *S. argus* | *Scatophagus argus* | NA | XM_046378215 | NA | NA | NA | XM_046379288 |
|  | *S. chuatsi* 1 | *Siniperca chuatsi* | NA | XM_044179737 | NA | XM_044205751 | XM_044194482 | XM_044189238 |
|  | *S. chuatsi* 2 | *Siniperca chuatsi* | NA | NA | NA | NA | XM_044194482 |  |
|  | *X. gladius* | *Xiphias gladius* | NA | XM_040135653 | NA | NA | XM_040133794 | XM_040146836 |
|  | *C. clupeaformis* 1 | *Coregonus clupeaformis* | NA | NA | NA | XM_041841106 | NA | XM_041865605 |
|  | *C. clupeaformis* 2 | *Coregonus clupeaformis* | NA | NA | NA | XM_041841102 | NA | XM_041865607 |
|  | *C. clupeaformis* 3 | *Coregonus clupeaformis* | NA | NA | NA | XM_041841095 | NA | NA |
|  | *T. rubripes* | *Takifugu rubripes* | NA | XM_003961323 | NA | NA | NA | DQ066526 |
|  | *P. kingsleyae* | *Paramormyrops kingsleyae* | NA | NA | NA | XM_023822193 | NA | NA |
| Outgroup | *B. splendens* 1 | *Betta splendens* | NA | XM_029135579 | NA | XM_029165763 | NA | M_041069420 |
|  | *B. splendens* 2 | *Betta splendens* | NA | NA | NA | XM_029165764 | NA | NA |
|  | *B. splendens* 3 | *Betta splendens* | NA | NA | NA | XM_029165765 | NA | NA |
|  | *A. testudineus* | *Anabas testudineus* | NA | XM_026350590 | XM_026347725 | XM_026358022 | NA | XM_033326154 |
|  | *C. lumpus* 1 | *Cyclopterus lumpus* | NA | XM_034558918 | NA | NA | XM_034535737 | NA |
|  | *C. lumpus* 2 | *Cyclopterus lumpus* | NA | NA | NA | NA | XM_034535738 | NA |
|  | *C. lumpus* 3 | *Cyclopterus lumpus* | NA | NA | NA | NA | XM_034535739 | NA |
