# Supplementary figures and images for: Spatiotemporal characteristics of the pharyngeal teeth in interspecific distant hybrids of cyprinid fish: Phylogeny and expression of the initiation marker genes
Source: Front Genet. 2022 Aug 16;13:983444. doi: 10.3389/fgene.2022.983444 (PMC9424816; doi:10.3389/fgene.2022.983444)

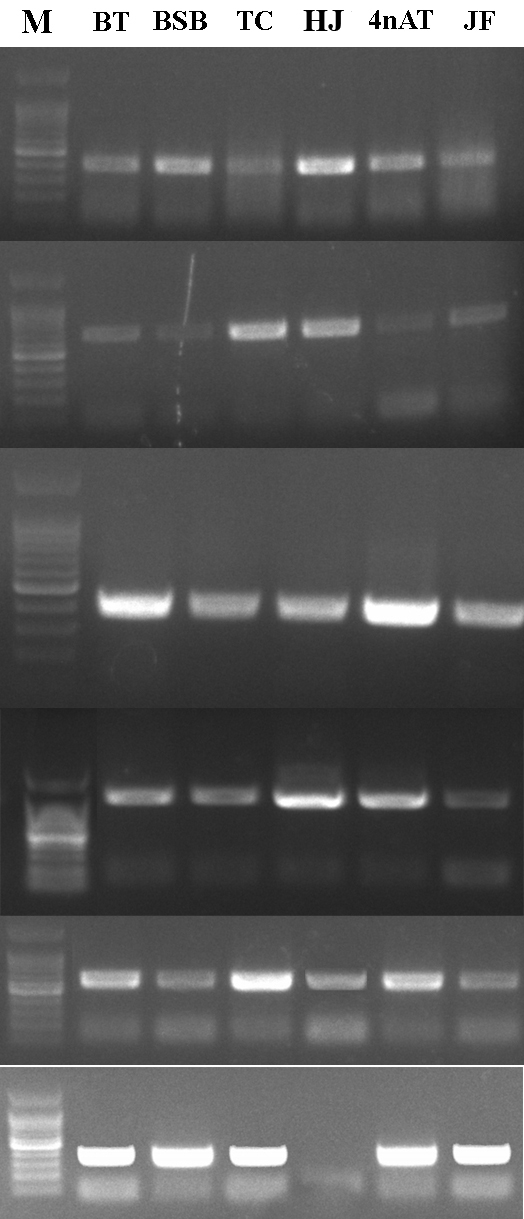

Supplement: Supplementary file 2 [file Image1.JPEG]
